# Prisms to Shift Pain Away: Pathophysiological and Therapeutic Exploration of CRPS with Prism Adaptation

**DOI:** 10.1155/2016/1694256

**Published:** 2016-09-07

**Authors:** Laure Christophe, Eric Chabanat, Ludovic Delporte, Patrice Revol, Pierre Volckmann, Sophie Jacquin-Courtois, Yves Rossetti

**Affiliations:** ^1^Service de Rééducation Neurologique, Pavillon Bourret, Hôpital Henry-Gabrielle, Hospices Civils de Lyon, 20 route de Vourles, Saint-Genis-Laval, France; ^2^Plateforme “Mouvement et Handicap” and Plateforme NeuroImmersion, Hôpital Henry-Gabrielle, Hospices Civils de Lyon, 20 route de Vourles, Saint-Genis-Laval, France; ^3^Inserm UMR-S 1028, CNRS UMR 5292, ImpAct, Centre de Recherche en Neurosciences de Lyon, Université Lyon 1, 16 avenue Lépine, 69676 Bron, France; ^4^Centre Orthopédique Paul Santy, 24 avenue Paul Santy, 69008 Lyon, France

## Abstract

Complex Regional Pain Syndrome (CRPS) is an invalidating chronic condition subsequent to peripheral lesions. There is growing consensus for a central contribution to CRPS. However, the nature of this central body representation disorder is increasingly debated. Although it has been repeatedly argued that CRPS results in motor neglect of the affected side, visual egocentric reference frame was found to be deviated toward the pain, that is, neglect of the healthy side. Accordingly, prism adaptation has been successfully used to normalize this deviation. This study aimed at clarifying whether 7 CRPS patients exhibited neglect as well as exploring the pathophysiological mechanisms of this manifestation and of the therapeutic effects of prism adaptation. Pain and quality of life, egocentric reference frames (visual and proprioceptive straight-ahead), and neglect tests (line bisection, kinematic analyses of motor neglect and motor extinction) were repeatedly assessed prior to, during, and following a one-week intense prism adaptation intervention. First, our results provide no support for visual and motor neglect in CRPS. Second, reference frames for body representations were not systematically deviated. Third, intensive prism adaptation intervention durably ameliorated pain and quality of life. As for spatial neglect, understanding the therapeutic effects of prism adaptation deserves further investigations.

## 1. Introduction 

Complex Regional Pain Syndrome is a usually lateralized chronic pain condition, which occurs following a potentially mild nociceptive event (trauma, surgery) or even without any triggering factor [1]. The pain is severe and disproportionate and is associated with reduced range of motion, loss of function, and disproportionate autonomic symptoms including edema and skin vasomotor alterations and trophic symptoms including osteoporosis [2]. Evolution of CRPS is usually long and unpredictable and most of the common therapies are only partially efficient [3]. Medical treatment is always associated with physiotherapy with the goal of maintaining range of motion while managing the level of pain (see [4], Moseley 2004 [5], and Bowering et al. 2013 [6]), before a second level of medical management implies more invasive interventions such as sympathetic nerve blockades [3]. For an important number of patients, beyond this level, no more therapeutic options are available while they still endure permanent and intense pain, with dramatic consequences for these patients. Beyond the chronic pain itself, their motor disability jeopardizes their job and social role, and they very frequently exhibit sleep perturbations and intense reactive depression, resulting in marked handicap and heavy social cost [7].

Although the precise mechanisms underlying CRPS may vary from individual to individual, including biological pathways that underlie aberrant inflammation or vasomotor dysfunction, it is generally accepted that, besides the peripheral pathology, maladaptive plastic processes in the central nervous system are crucially involved [2, 7]. Indeed, it is widely acknowledged that body representation abnormalities are observed in CRPS patients: impaired laterality recognition of the affected limb [8–10] or lateral bias of the visual subjective body midline (vSM) [11, 12]. Among the reported symptoms, the affected limb can be felt bigger than it actually is [9], patients can have strangeness feeling about their limb [13], or the limb can be felt like not belonging to the patient anymore or needing exaggerated attention to be moved or even unusual effort to be integrated in the body schema [14].

One of the most common and disabling symptoms is underuse of the affected limb, which has been related to a kind of motor neglect, described by Galer et al. [15]: movements performed by the affected arm were clinically described as hypokinetic, bradykinetic, and hypometric. They chose the term of “neglect-like” to qualify these deficits, leading to an ever-increasing amount of publications speculating on the parallel between spatial neglect following stroke and disturbance of body representation exhibited by CRPS patients [11–14, 16–18, 18–20]. Indeed, spatial neglect includes a variety of symptoms and one of the most striking is perceptual neglect: difficulties to detect, respond to, or orient their attention toward stimuli presented on the left side of space [21]. For example, patients would not eat the left side of their plate, omit to make up or to shave the left side of their face, hurt their left arm when passing through doorways, or exhibit less auditory attention to their left side [22]. Despite the fact that CRPS patients have no brain lesion and obviously do not show such a severe attentional bias, the parallel between these two syndromes has been repeatedly drawn, leading to publications investigating perceptual neglect symptoms in CRPS. This hypothesis has been explored with specifically designed subjective questionnaires [13, 14, 16, 18] or more objective clinical examinations [11–13, 18–20, 23, 24]. Altogether, these reports provide contradictory or incomplete data about spatial cognition of CRPS patients. For example, Sumitani et al. [11] found a deviation of visual straight-ahead toward the painful side (which is opposite to the neglect hypothesis) while Reinersmann et al. [12] argued that this measure was deviated toward the left side irrespective of the CRPS side. Förderreuther et al. [13] showed a deviation of line bisection toward the painful side only in right CRPS performing the bisection with their healthy hand but not in left CRPS. Moseley et al. [23] showed spatially defined attentional shift in tactile processing (being slower on the hand placed in the affected side of space). Reid et al. [24] suggested a body centred spatial inattention pattern, which was modality specific or only affected if testing involved bodily relevant stimuli. Another hypothesis concerning CRPS patients' spatial cognition is that it consists of motor rather than perceptual neglect [25, 26]. This idea is fully compatible with the initial finding that patients with CRPS exhibit underuse of their affected limb [14], but it has not been formally explored. To sum up, while there is a growing agreement that CRPS is more than a peripheral disorder (e.g., [3, 7, 11, 12, 27]), there is absolutely no available consensus about what the central contribution to CRPS might be, which type of neglect it might relate to, and which side of space should be neglected with respect to the lesioned limb [11, 14, 24, 26, 28].

Another very promising and intriguing perspective concerning CRPS is prism adaptation therapy. This proposal was also based on similarities between spatial neglect and CRPS [29]. As a matter of fact, this deceptively simple technique has proven to be very efficient for manifestations of neglect and visuospatial disorders [30–34] and is noninvasive. Sumitani et al. [11] discovered that visual straight-ahead demonstrations performed by CRPS patients were biased toward their affected side. They accordingly used prism adaptation to improve this spatial cognition bias and were the first to describe the effect of prism adaptation on CRPS. They used prisms inducing an optical shift away from the painful side and observed significant release of pain in 5/5 patients [29]. This result was later replicated on one patient by Bultitude and Rafal [35]. It is interesting to note that Sumitani et al. [29] also performed a compelling longitudinal follow-up on one patient, indicating the directional specificity of prisms. Indeed, neutral prisms did not alter pain while prisms inducing visual bias toward the painful side tended to increase pain. To explain this therapeutic effect, it has been proposed that the attentional bias toward the painful side would enhance the weight of nociceptive over epicritic stimuli and consequently contributes to maintaining pain [36]. Another hypothesis is brought about by Reid et al. [24]. According to them, pain favors a tendency to protection, increasing visual scanning, which would explain the visuospatial bias toward the painful side during immobilization while compensatory overuse of the contralateral limb would be responsible for the processing bias away from the painful side for the body-relevant stimuli. As a matter of fact, using prism adaption toward the healthy side is known to produce visual aftereffects toward this side and manual aftereffects toward the painful side. The latter effect should reduce the initial bias found by Sumitani et al. [29] and subsequently reduce pain.

This analysis of the available literature can be summarized in 3 main points. First, the parallels and the differences between neglect patients and CRPS patients need to be specifically investigated. Second, prism adaptation seems to be a promising method to alleviate CRPS patients, but to our knowledge only two studies are available and insufficient data is available about follow-up of these patients. Sumitani et al. [29] followed up only one patient for 6 months with various types of prims adaptation periods and a two-month-long wash-out period; and Bultitude and Rafal [35] mentioned that, after 13 days after the end of the treatment, their patient's pain resumed. Currently available data is insufficient to propose prism adaptation as an evidence-based intervention to alleviate CRPS pain. It is also too scarce to elaborate a large double-blind therapeutic essay. So far, there is no available report about the efficacy of prism adaptation at the functional level. It is also important to estimate what the prism adaptation posology is which may lead to both significant and sustainable therapeutic gain. Third, given the strong discrepancies found in the literature, straight-ahead demonstrations need to be monitored in more detail in CRPS before and during prism adaptation, so as to provide a better understanding of the mechanisms underlying prism adaptation efficiency on pain.

The main objective of the present study was to investigate the characteristics of spatial cognition alterations presented by CRPS patients. To do so, we explored different aspect of spatial neglect before and after a prism adaptation therapy. Perceptual aspects were explored with classical paper-and-pencil neglect tests as well as not only visual but also manual straight-ahead demonstrations. Motor aspects (extinction or “neglect-like” deficits) were explored using two specific tasks monitored by quantitative motion analysis. Our second objective was to explore the efficiency of prism adaptation rehabilitation on CRPS pain on a larger sample and with a longer follow-up than what was previously published, with a special emphasis on quality of life in order to assess the functional potential of this rehabilitation technique. We used a posology of two prism adaptation sessions per day in order to optimize the duration of aftereffects. Our third objective was to monitor the evolution of visual and manual straight-ahead pre-, per-, and postprism adaptation in order to investigate their dynamical relationship and explore their potential causal links.

## 2. Material and Methods

### 2.1. Patients

Seven consecutive patients reffered from pain or postsurgery consultations and suffering from CRPS following a surgery or a traumatic event were included in the study from January 2014 to October 2015. All of the patients fulfilled the Budapest criteria for the diagnosis of CRPS and provided written informed consent to participate in the study. Two additional patients were excluded because of conversion etiology or pulling out.

The inclusion criteria were age ranging from 18 to 90 years; CRPS type 1 or 2 concerning the hand, wrist, and forearm; traumatic or surgical etiology; chronic evolution of longer than 2 months. The exclusion criteria were severe psychiatric disorder; ongoing sympathetic nerve blockade cycle; inclusion in another interventional study regarding CRPS.

The patients' characteristics are detailed in Table 1.

### 2.2. Study Description

**Figure 1:**
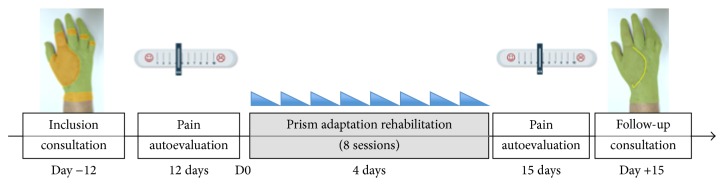
Global time chart. Patients were seen at around day −12 for inclusion, and then pain autoevaluation was performed during a period of about 12 days, followed by 4 days of prism adaptation and then pain autoevaluation during a period of about 15 days, and they were then seen at follow-up consultation.

This is an open, monocentric, interventional, usual care study involving a single group of patients who all underwent prismatic adaptation rehabilitation.  Figure 1 summarizes the study design. The main objective of this study was to qualify and quantify neglect-like symptoms before intervention and their evolution during repetitive prism adaptation therapy. The secondary objective was to evaluate the efficiency of PA rehabilitation on CRPS symptoms. For this purpose, we used the level of pain as the primary outcome measure and the Sickness Impact Profile as the secondary outcome measure. To this aim, we monitored spatial reference examination, which included visual straight-ahead, manual straight-ahead, open loop pointing (to assess aftereffects), and line bisection task. We also looked for motor extinction and/or motor neglect with a 3D kinematic movement analysis by means of a finger tapping task and a circle drawing task recorded before and after prism adaptation rehabilitation. A control group of 6 healthy subjects allows comparison between CRPS patients before intervention regarding previously unquantified kinematic tests.

### 2.3. Study Parameters

#### 2.3.1. Pain Monitoring

Pain was measured using two complementary ways. First, at the inclusion and follow-up consultations and before and after each prism adaptation session, we used the basic VAS (Visual Analog Scale). Second, when the patients stayed home between inclusion and rehabilitation and then between rehabilitation period and follow-up consultation, they collected by themselves their level of pain using adapted VAS: on a ten-centimeter-long line, they marked their level of pain using the same conventions as for VAS, twice a day during a period of 10 days (in the morning and in the evening). The patients were asked to hide each previous line in order to avoid a possible bias. Patients brought the sheet back at the consultation and the examiner measured each line to the nearest millimeter, resulting in values between 0 and 100, as for the classical VAS. Data was analyzed using regression analysis for preadaptation values and two-way ANOVA and repeated measures ANOVA with planned comparisons for the follow-up values.

For the last included patient, we followed the idea of a previous patient and asked her to visually represent her pain on her hand picture every treatment day and at inclusion and follow-up consultations. Drawing and coloring were performed on real-size pictures of her own hand (palm and back) using a simple color code. Green was used to represent the skin areas with no pain, yellow was used for light pain, orange was used for moderate pain, and red was used for intense pain. This procedure provides a vivid way to better follow up pain evolution during treatment.

#### 2.3.2. Sickness Impact Profile

A questionnaire was given to patients before the beginning of rehabilitation and at the beginning of the follow-up consultation [37]. This data set was analyzed with two-way ANOVA.

#### 2.3.3. Prism Adaptation

PA was carried out by wearing a pair of glasses (http://OptiquePeter.com/) producing a 15° optical deviation of the visual field toward the healthy side of the body [29]. The prismatic lenses were composed of two superimposed, curved, point-to-point lenses fitted with a “glacier” frame containing lateral leather protectors designed to avoid access to nonshifted vision. During prism exposure, the patients were invited to execute 80 rapid pointing movements toward visual targets located 10 degrees to the left or to the right of the body middle, in a pseudorandom order, as in neglect studies [38, 39]. Our patients were all able to follow the instructions to carry out rapid movements and large errors were observed at the end of their first pointing movements, leading to actual sensorimotor adaptation. They obviously were aware of the visual shift, unlike neglect patients. Each patient underwent 8 prism adaptation sessions, at the frequency of two sessions per day, separated by at least five hours (e.g., 9 a.m. and 2 p.m.). Patients performed prism adaptation with their affected hand, except for one for whom the repeated movements were too painful.

#### 2.3.4. Visual Straight-Ahead

Patients sat comfortably with their head on a chin rest, facing straight a screen that was either 100 cm or 200 cm ahead. The experiment was carried out in total darkness. A small red dot (LED) appeared at eye level at approximately 30° alternatively to the right or the left of the objective body midline (OM). This red dot was moved from right to left or vice versa at about 3°/second. The participants were asked to stop the dot, using verbal command, when the position crossed their midsagittal plane (vSM). The vSM position was computed between vSM and OM in degrees of visual angle. A rightward deviation was signed positive while a negative value indicates a leftward shift. Ten trials for each condition (100 cm and 200 cm) were performed at inclusion, before and after each prism adaptation session, and at follow-up consultation. Mean and standard deviation were computed for each session and condition.

#### 2.3.5. Manual/Proprioceptive Straight-Ahead

The patients were comfortably seated in front of a table. A chin rest maintained the trunk in an upright position and the head straight. The patients were asked to point in darkness at the “straight-ahead” position, that is, in the direction of an imaginary line dividing their body into two equivalent halves. The patients spread out their arm without any speed or amplitude constraint. The patients wore a metallic thimble on their index finger. When the finger touched the table surface covered with carbon isoresistive paper, tension between the thimble contact point and the reference electrode was measured (see [39]). Then, the angular position (in degrees) relative to the objective sagittal axis was computed and conventionally signed (negative on the left, positive on the right). Measurement precision was ±0.5 degrees. Ten trials were performed with each hand at inclusion, before and after each prism adaptation session, and at follow-up consultation.

One of our predictions was that MSA might be deviated toward the painful side. As there were 5 right CRPS and 2 left CRPS, we normalized the data so as to obtain two groups of means. First, we pooled the data on the basis of the affected and healthy hand. In order to obtain homogeneous values, we logically also used the opposite value of the left CRPS data (the sign of each value was changed).

#### 2.3.6. Open Loop Pointing

Open loop pointing (OLP) accuracy measurement was carried out under the same conditions of darkness and with the same devices. The luminous visual target was aligned with the patients' sagittal axis. The instruction given to the patients was to place their index finger (right and left hand) at the target drip-line as precisely as possible without time constraint. Data collection and processing were similar to MSA.

#### 2.3.7. Line Bisection Task

The patients were seated in front of a table, with an A4 sheet of paper lying on the table and aligned with their body axis on which a centered 200 mm long and 2 mm thick line was printed. The patients were asked to mark the middle of the line without any computation or external help. The distance was calculated by measuring the shift in millimeters between the reported point and the objective midline. A leftward shift had a negative value and a rightward shift had a positive value. Ten measures were obtained with each hand at inclusion, before and after each prism adaptation session, and at follow-up consultation. We used the same method described for manual straight-ahead to obtain two groups of normalized data: affected and healthy hand, based on right CRPS group.

#### 2.3.8. Statistical Analysis

The two pretests for spatial parameters (visual and manual demonstrations and line bisection acquired at inclusion and during pre-ADA1) were used to explore their reliability by means of correlation. In order to investigate whether left and right CRPS patients would exhibit left or right biases, Yates Chi-2 tests were performed for each parameter.

## 3. Motor Extinction Tasks in Kinematic Analysis

### 3.1. Finger Tapping Task

Subjects sat in a comfortable armchair facing a table, with hands lying on the table with the palms down. In this resting position, they were blindfolded and asked to listen to a metronome sound at 120 beats per min during a period of 10 seconds and to remember it. After a go signal given by the experimenter, they were asked to tap with their index finger on the table at the previously heard frequency during a period of 30 seconds. This tapping test was performed in three different conditions. In the “opened-eyes” and the “closed-eyes” conditions, the two hands were 28 cm apart and positioned symmetrically on their side. In the “crossed hands” condition, eyes were closed and the right hand was positioned over the left hand for the first run and under the left hand for the second run.

For each condition, movements were recorded using three different blocks in the following order: right index alone, left index alone, and both indexes simultaneously. This series of three blocks was repeated twice so that two runs were collected for each combination of condition and block. Thus, each participant performed a total of 18 trials (3 conditions × 3 blocks × 2 runs).

We used an optoelectronic Vicon MX Giganet system composed of eight infrared stroboscopes and 100 Hz infrared cameras to record 3D motion of passive markers during the finger tapping test. One passive infrared reflecting marker was placed upon the nail of the two index fingers. Each data acquisition began with the go signal given by the experimenter and ended automatically after 30 seconds. After recording and tridimensional reconstruction, the spatial positions of each marker were filtered using a Butterworth low-pass filter at 6 Hz cut-off frequency. The spatial position of the index nail marker was used to compute the relevant movement kinematic parameters: movement amplitude and time intervals between taps (i.e., period).

For each condition and for each patient, a mean value was computed for each individual tap using the two runs so as to respect a potential extinction effect along the task. Further analyses used this series of averaged values over the two runs.

The data was analyzed with repeated measures and two-way ANOVA (uni/bimanual condition *∗* left/right hand). Controls and patients data were analyzed separately because of variance inhomogeneity for this task.

### 3.2. Circle Drawing Task

**Figure 2:**
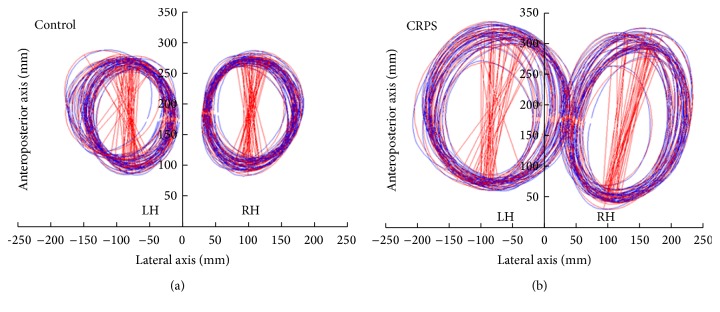
Circle drawing task. Example of circle drawing task for a control (a) and a patient (b). LH: left hand; RH: right hand. The blue tracks depict the actual drawing performed by the subject with the left hand (LH) or right hand (RH), whereas the red tracks represent the ellipses fitted to the drawing. The red straight lines depict each ellipse main axis, showing a limited variability in orientation and size. Note that the neglect-like prediction that the left affected hand produces smaller circles is not verified in this representative patient.

Patients sat in the same position as previously described. An A3 sheet of paper was positioned on the table and aligned with the body axis and two reference points distant by 5 centimeters (each 2.5 cm from the sagittal axis) were printed on it. The patients held a pen in each hand and were asked to listen to a 60/min beat throughout each trial. They were requested to simultaneously draw circles with each hand, crossing the reference point at each beat. Three conditions were recorded: full vision, that is, eyes open and pen on (double visual feedback); hand vision, that is, eyes open and pen with cap (single visual feedback); and no vision, that is, eyes closed (no visual feedback). In each condition, clockwise circles were drawn with the left hand and counterclockwise circles were drawn with the right hand simultaneously in a block and vice versa in a second block. Instruction was to draw large circles, filling most of the available space on the page.  Figure 2 shows an example of this task for a patient and a control.

So each participant performed a total of 6 trials (3 conditions × 2). We used the same optoelectronic Vicon MX Giganet system. Data acquisition and reconstruction were identical to the previous task. Two passive infrared reflecting markers were placed over the pen lead and on the cap, depending on the condition. The spatial position of the pen marker was used to reconstruct ellipses drawn by the subjects.  Figure 2 shows the actual and fitted ellipses produced by each hand by a patient and a control subject.

Customized software was used to compute the ellipses characteristics: perimeter, angle, center coordinates, horizontal extent (depicting their maximal width along the horizontal axis), surface, and horizontal drift and its direction. The most relevant parameters to compare ellipse size produced by each hand were therefore ellipse perimeter, surface, and horizontal extent. In addition, the horizontal drift of the ellipses was examined, that is, the most relevant dimension to explore neglect-like behavior.

This data was analyzed with three-way ANOVA and (pre-post*∗*condition*∗*hand). For this task, comparisons between controls and patients were possible thanks to the comparable standard deviation in both groups with three-way ANOVA (group*∗*condition*∗*hand).

In the relevant figures, standard error of the mean (SEM) is represented with error bars.

## 4. Results

### 4.1. Initial Pain and Functional Assessment

Concerning the pain level before intervention, it was important to check whether the level of pain was stable before prism adaptation. For none of the patients, the individual analysis of regression on pain level over time showed a significant slope (all *R*'s < 0.58; all *p*'s > 0.14). Therefore, no preintervention trend would interfere with potential amelioration of this symptom following the intervention. The mean level of pain before intervention was 58.8 ± 12.6 on the VAS the day before the beginning of intervention and significantly differed from zero (*t*(6) = 12.3; *p* < 0.0001).

The mean initial Sickness Impact Profile for the patients was 38.7 ± 15.3 points (the nearest to zero the score, the better the quality of life) and significantly differed from zero (*t*(6) = 6.72; *p* < 0.001), indicating a significant impact on quality of life.

### 4.2. Spatial Body Frames of Reference

**Figure 3:**
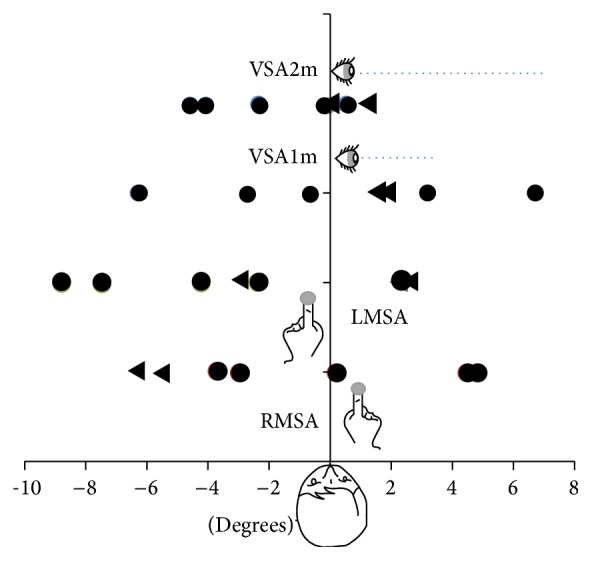
Initial reference frame evaluation for each patient (measured at inclusion), in degrees of angle deviation from objective midline. No systematic bias was found in terms of CRPS side: most variables are fairly symmetrically distributed around the sagittal axis. The only significant trends were found for the healthy hand straight-ahead demonstration which was biased toward the left for both left (RMSA) and right (LMSA) CRPS. RMSA: manual straight-ahead for the right hand. LMSA: manual straight-ahead for the left hand. VSA1m: visual straight-ahead at 1 meter. VSA2m: visual straight-ahead at 2 meters. Circles: right CRPS. Triangles: left CRPS.

Before adaptation, spatial reference frames deviation in CRPS was explored twice (inclusion consultation (Figure 3) and pre-ADA1 measures) with six parameters (visual straight-ahead at one meter (VSA1m), visual straight-ahead at two meters (VSA2m), manual straight-ahead for the right hand (RMSA), manual straight-ahead for the left hand (LMSA), and line bisection for each hand (RLB and LLB)). These measurements allowed us to test for the reliability of these sensorimotor measurements. No significant correlations between test and retest for VSA1m (*F*(1,5) = 0.01; *p* = 0.96), VSA2m (*F*(1,5) = 0.12; *p* = 0.76), and LMSA (*F*(1,5) = 0.14; *p* = 0.72) were found. The best reliability was found for RMSA (*F*(1,5) = 2.92; *p* = 0.15). Average values obtained for right and left CRPS patients are presented in Table 2.

In order to investigate whether left and right CRPS patients would exhibit left or right biases, Yates Chi-2 tests were performed for each parameter. None of them provided significant results (Yates corrected Chi-2: 0.36, *p* = 0.55 for VSA1m, VSA2m, and RMSA, and 0.02, *p* = 0.89 for LMSA). Altogether, we conclude that no systematic bias of reference frame was obtained in our patient sample.

Line bisection before adaptation tended to be overall accurate: the affected hand performed with a 0.5 ± 0.4 mm bias and then 1.6 ± 0.5 mm bias at the second test, and the healthy hand showed 0.7 ± 0.3 mm bias and then −1.0 ± 0.3 mm bias at the second test. None of these deviations were statistically significant (all *t*(5) < 1.35; all *p* > 0.25).

### 4.3. Kinematic Analyses

As presented above, another hypothesis concerning CRPS patients was the presence of motor neglect and/or motor extinction. To explore this question, patients underwent circle drawing and finger tapping tasks.

Concerning* the finger tapping task*, the main objective was to assess whether patients presented motor neglect, that is, poorer performance with the affected hand in the unimanual condition, and motor extinction, that is, poorer performance for the affected hand in the bimanual condition. The most relevant parameters to evaluate performance were amplitude and the time between two taps. Logically, the most relevant condition to bring to light motor extinction or neglect should be the closed-eyes condition. We also used a crossed hands condition so as to explore a potential effect of space if motor neglect was found.

Means for the three conditions for controls and pre- and postadaptation patients are presented in Table 3. We will describe in detail here only the reference closed-eyes condition for patients and controls and the comparison between the two groups. Comparisons between patients before adaptation and controls were conducted using three-way ANOVA (group*∗*uni/bimanual  condition*∗*hand), as shown in Table 4. Crucially, no main effect of group was found in the no-vision reference condition (amplitude: *F*(1,9) = 1.18; *p* = 0.3; period: *F*(1,9) = 1.16; *p* = 0.3). Crucially, no group*∗*hand interaction was found for amplitude (*F*(1,9) = 0.35; *p* = 0.57) or for period (*F*(1,9) = 2.46; *p* = 0.15). This means that, before adaptation, patients did not differ from controls in terms of tapping performance which is crucial because it stands in sharp contrast with the neglect prediction.

*The circle drawing task* further explored motor extinction and motor neglect. Indexes of ellipse size (perimeter, surface, and horizontal extent) investigated whether patients performed smaller circles with their affected hand, that is, motor extinction. Horizontal drift measured the difference between the last and first circle drawn, and the direction of this drift would provide an argument for neglect if it was congruent between the two hands. For this task, the most likely condition to exhibit extinction or neglect was again the closed-eyes condition as no visual feedback is provided.

**Figure 4:**
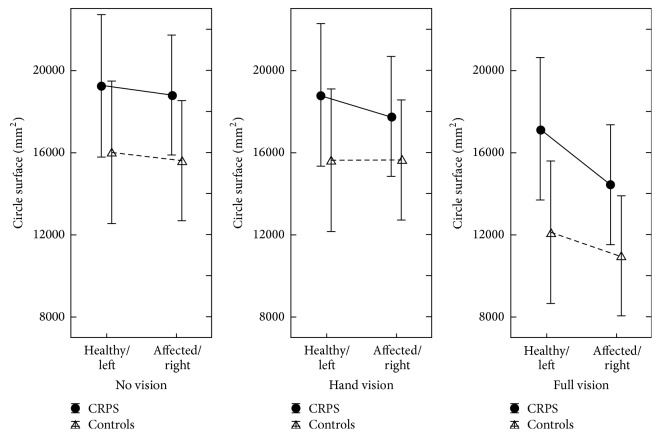
Circle drawing task for controls and CRPS patients in the three conditions (no vision, hand vision, and full vision) and for the two hands (left and right for controls, healthy and affected hands for patients). Overall CRPS tended to perform larger circles than controls. The more the visual feedback was available, the smaller the circles were for both controls and patients. A tendency to asymmetry between the two hands was paradoxically observed when vision was available in both groups. Errors bars depict the standard error of the mean.

The most obvious result in Figure 4 is that patients draw larger circles than controls. Both patients and controls tended to produce larger circles with their left hand in all conditions. In the reference no-vision condition, the left-right ratio obtained for the average circle surface was identical in patients and controls (i.e., 2.5%) as shown in Tables 6 and 7. Another tendency visible in Figure 4 is that the more the vision available to the subjects, the smaller the circle. In addition, with more vision, the drawing drifted less apart and the difference in circle size between the two hands diminished. The detailed analysis of this data set showed a reliable drawing direction effect on ellipse size parameters (perimeter, horizontal width, and surface), which was not relevant to the aim of the current study (Tables 6 and 7). A significant hand effect logically affected the horizontal drift (the two hands symmetrically drifting apart in order not to bump each other). The ellipse main axis angle was also logically affected by a hand × direction interaction as a result of biomechanical constraints. More pertinently, no group effect was observed, either as main or as interaction effects.

### 4.4. Prism Adaptation

**Figure 5:**
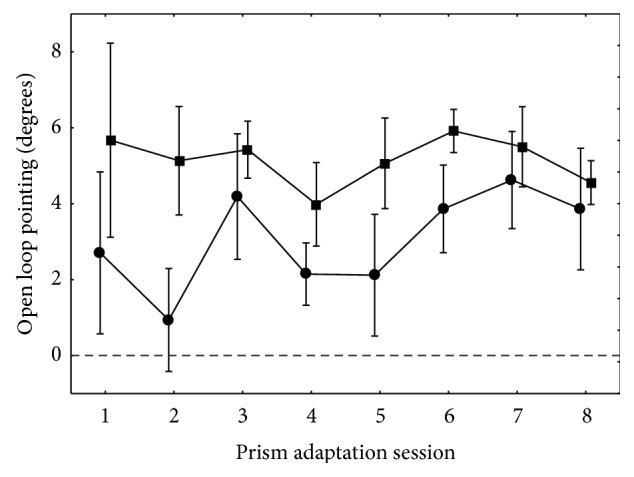
Open loop pointing (±SEM) for the adapted hand throughout the prism rehabilitation period. This figure displays preadaptative (circles) and postadaptative (squares) values of open loop pointing movements performed with the adapted hand toward a visual target. The difference between the two curves shows that every session gave rise to the expected compensatory aftereffects. Although the posttest seemed to remain fairly stable over time, indicating that a maximal shift in OLP was obtained from the first session, the gradual shift of the pretest values suggested that some retention of aftereffects was gained and capitalized between each session. Therefore, the apparent size of aftereffects appears to reduce with time, even if the ANOVA interaction between session and the pre-post effect did not reach significance.

During the prism adaptation period, the most relevant parameter to evaluate the reality of adaptation is the presence of aftereffects. These aftereffects are quantified by open loop pointing performed with the adapted hand (AHOLP). Concerning this parameter, the repeated measures ANOVA (session*∗*pre-post) on the pre- and postvalues for each session showed a main effect of adaptation (i.e., pre- versus postmeasures (pre-post)) (*F*(1,6) = 16.56; *p* < 0.01). As can be seen in Figure 5, each adaptation session produced compensatory aftereffects toward the right side. Postadaptation values seemed to remain stable throughout the series of 8 sessions (*y* = −0.04*x* + 5.38; *t*(6) = −0.40; *p* = 0.71), but the preadaptation values tended to drift gradually toward the right (*y* = 0.54*x* + 0.28; *t*(6) = 3.0; *p* < 0.03). This suggests that the effect of an adaptation session was partially retained until the next session. Although the magnitude of these effects seems to decrease over time, there was no significant interaction between pre-post and session (*F*(7,42) = 0.89; *p* = 0.53). Despite the evolution of pretest values, planned comparison between the inclusion and follow-up consultation values was not significant (*F*(1,6) = 0.15; *p* = 0.71), suggesting that the modification of the open loop pointing did not fully stabilize and at least partially resolved in between the end of the treatment and the follow-up consultation.

As in most experiments, the aftereffects obtained with the adapted hand did not transfer to the nonadapted hand, as shown by the repeated measures ANOVA (session*∗*pre-post) on the open loop pointing which showed no significant main effect of pre-post (*F*(1,6) = 1.98; *p* = 0.21) or interaction (*F*(7,42) = 0.39; *p* = 0.90).

Other parameters can be classically modified by prism adaptation: the visual straight-ahead at one meter (VSA1m), visual straight-ahead at two meters (VSA2m), manual straight-ahead for adapted hand (AHMSA), and manual straight-ahead for nonadapted hand (NAHMSA). The same analyses were performed as for the OLP for these four parameters and showed only marginally significant effect of pre-post for VSA1m (*F*(1,5) = 7.76; *p* = 0.06) and marginally significant effect of the session*∗*pre-post interaction (*F*(7,42) = 2.2; *p* = 0.053). The session*∗*pre-post  interaction was significant for NAHMSA (*F*(7,42) = 2.74; *p* < 0.02) which means the aftereffects amplitude varied. For none of these four parameters, the planned comparisons between inclusion and follow-up consultation were significant.

### 4.5. Expansion of Prism Adaptation

Finally, several variables addressed the clinical purpose of this study: the efficiency of prism adaptation beyond the adapted sensorimotor function. The effects of prism adaptation therapy were assessed on clinical variables, pain and quality of life, as well as on line bisection and circle drawing. Regarding the level of pain, our analyses attempted to answer the following questions: did each session of prism adaptation increase pain because of the painful hand solicitation? Did prism adaptation release pain?

**Figure 6:**
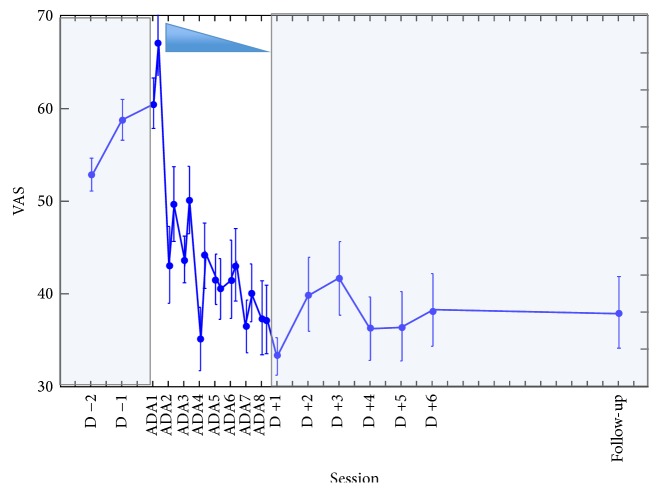
Evolution of mean pain (±SEM) for the 7 patients from 2 days before prism adaptation to follow-up consultation. In white background is the prism rehabilitation period. The shaded background indicates the pre- and postperiods of follow-up. Before the intervention, the apparent increase of average pain level was not confirmed by individual analyses. During the intervention period, a substantial decrease of pain is observed. Following the intervention and up to the follow-up consultation (D +15), remarkable stability of pain measures was observed.

Concerning the evolution of pain during the period of prism adaptation, two-way (session ∗ pre-post) ANOVA was realized on VAS values during the week of intervention (i.e., 8 pairs of pre- and postvalues for each patient). This analysis showed a highly significant main effect of session (*F*(7,42) = 4.77; *p* < 0.0006). As shown in Figure 6, the pain level specifically decreased during the period of adaptation. Importantly, no main effect of pre-post was observed (*F*(1,6) = 2.58; *p* = 0.16), which implies that the affected hand solicitation during each session of prism adaptation did not increase pain in a short term.

Finally, repeated measures ANOVA performed on the whole pain data available (pre-, per-, and postprism adaptation, including the pain level collected at the follow-up consultation) was highly significant (*F*(24,144) = 3.42; *p* < 0.0001) showing that the level of pain during the whole observation period was not stable. Then, planned comparisons allowed us to further specify the timing of pain amelioration. A comparison between pre- and peradaptation values showed a significant difference between these two groups (*F*(1,6) = 7.92; *p* < 0.05) showing that pain substantially diminished during and after prism adaptation. Then, a comparison between the last adaptation session (ADA8) values and all the postadaptation values showed no significant difference (*F*(1,2) = 3.98; *p* = 0.18), implying that pain benefit remained stable over the follow-up period. Congruently, comparison between pre- and postintervention level of pain was significant (*F*(1,6) = 14.15; *p* < 0.01), as well as preadaptation values compared to the follow-up consultation pain level (*F*(1,6) = 12.31; *p* < 0.02) further supporting the reliability of the benefit over time.

**Figure 7:**
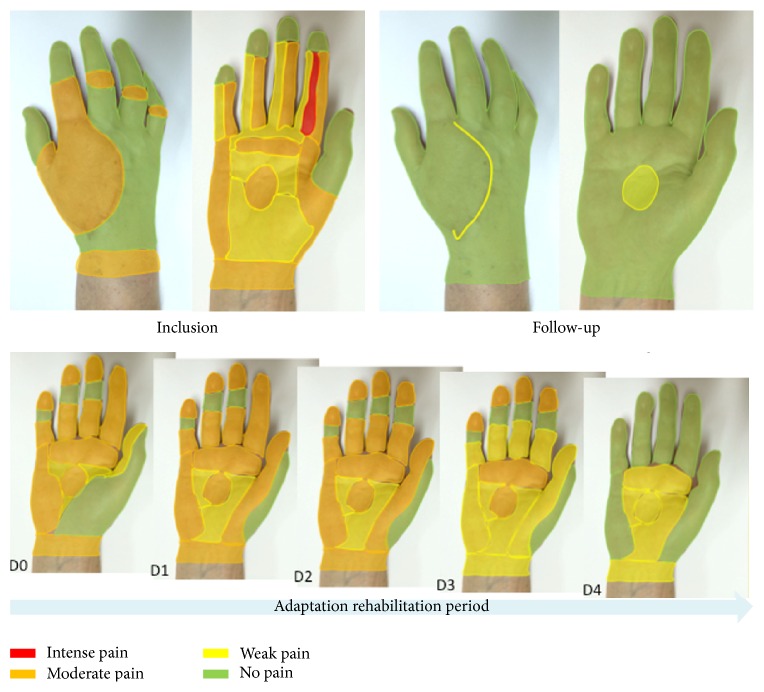
Visual representation of pain evolution for one patient. This patient developed CRPS following a trauma in a car accident with no fracture but some tissue lost on her hand back for which she had a skin graft. The initial mapping of pain (inclusion) was surprisingly nonmonotonous, with idiopathic design of pain intensity areas, and predominantly involved the palm even though this side was not concerned by surgery. The evolution of pain evaluation is depicted from the first (D0) to the last (D4) day of the intervention. A demonstrative reduction of surface and intensity of pain was observed over all hand territories. This pain mapping in space and time allows observing that the most painful territories do not recover last and that there is no clear anatomofunctional rationale for the shape and size of individual areas or for their temporal evolution. At the follow-up consultation, a nearly normalized mapping was produced by the patient, except for a tiny area in the palm and a portion of the hand back side's scar.

Incidentally, the last recorded patient was asked to visually represent her pain on her own hand picture (palm and back) with a simple color code (Figure 7). Qualitatively, her drawings enabled us to precisely track the evolution of pain sensations for each skin territory. As represented in Figure 7, the surface and intensity of pain dramatically decreased along the prism adaptation period. But maybe the most surprising effect is the further improvement after the end of prism rehabilitation, which indicates that plasticity effects went on even after the end of the prism rehabilitation period.

**Figure 8:**
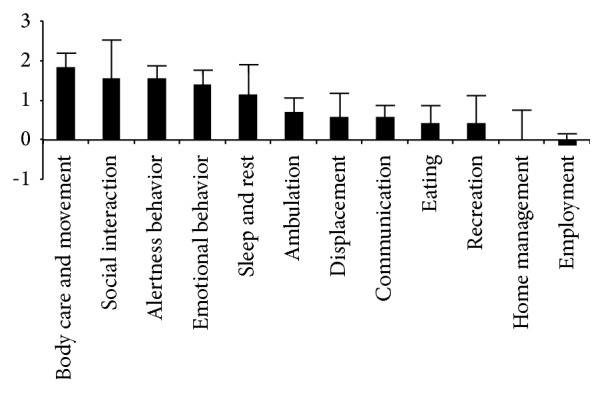
Improvement of subscores for each SIP category (±SEM). Positive values indicate improvement in quality of life. The largest improvements are observed for body care and movement, social interactions, alertness behavior, and emotional behavior. Each column represents the mean differential value between the pre- and postintervention measures (whiskers represent standard error of the mean).

On the Sickness Impact Profile scale, the two-way (category, pre-post) ANOVA showed a main effect of category (*F*(11,66) = 5.61; *p* < 0.0005) which is simply congruent with the fact that the different categories of this score do not include the same number of items. More importantly, a main effect of pre-post was also observed (*F*(1,6) = 8.2; *p* < 0.05). The preglobal score was 38.7 ± 5.76 points (mean ± SEM) while postscore was only 28.6 ± 4.64 points, that is, resulting in an improvement of 10 points on the SIP scale. This result demonstrates a substantive improvement of quality of life following the intervention. The interaction between category and pre-post was only marginally significant (*F*(11,66) = 1.83; *p* = 0.066). The most improved SIP subscores corresponded to emotional behavior, social interactions, body care and movement, and alertness behavior, as shown in Figure 8.

Interestingly, no significant correlations were found between the ADA1 posteffects on AHOLP and the global efficiency on pain (*F*(1,5) = 0.05; *p* = 0.83) or on SIP (*F*(1,5) = 0.04; *p* = 0.85).

*Line bisection* was not significantly altered during the pretest. Following prism adaptation, the average values obtained with the right and the left hand tended to be stable compared to pretest ones. The three-way (hand, session, pre-post) repeated measures ANOVA showed only a marginal effect of session (*F*(7,28) = 2.24; *p* = 0.061). Surprisingly, we did not find a main effect of pre-post (*F*(1,4) = 2.65; *p* = 0.18).

In the* finger tapping task*, patients did not significantly differ from controls in terms of initial hand asymmetry (Table 4). Nevertheless, this study offered the opportunity to test whether prism adaptation therapy affected this initially unperturbed parameter in CRPS patients. As depicted in Table 5, no significant result emerged from this analysis. Specifically, no effect of prism adaptation (pre-post) reached significance and no significant interaction emerged. Interestingly, this applies to both unimanual and bimanual conditions.

In the* circle drawing task* too, patients did not significantly differ from controls in terms of initial hand asymmetry (Table 7). Following prism adaptation, only hand and direction effects reached significance (Table 8), confirming the main effects found during the pretest in both subjects groups (Table 7). The hand effects tended to be prominent in the full-vision condition whereas the direction effects tended to be prominent in the no-vision condition (Table 7). Only one pre-posteffect nearly reached the significance level: the surface of the circles drawn after prism adaptation tended to be larger in the full-vision condition. Crucially, no significant pre-post *∗*hand interaction was obtained, which means that the hand difference observed during the pretest in patients and controls was not affected by the prism adaptation therapy.

## 5. Discussion 

This study addressed three main issues. First, we shed new light on CRPS patients' spatial cognition alterations by investigating perceptual neglect, motor neglect, and motor extinction before intervention. Second, we explore the potential effect of prism adaptation rehabilitation on CRPS pain in terms of consequences on quality of life and of duration following a week of intense treatment. Third, we monitor the evolution of perceptual neglect from pre- through to posttreatment phase in order to explore the dynamical relationship between spatial cognition, pain, and functional effects of the treatment and better understand their potential causal links.

### 5.1. Spatial Cognition in CRPS: Testing the Neglect Hypothesis

Our first goal was to explore different aspects of spatial cognition in order to explore whether CRPS patients present neglect-like characteristics: which subcategories of spatial cognition were potentially impaired and how these classic neglect parameters would evolve with prism adaptation. Perceptual neglect was assessed with sensitive and quantitative neglect tests, namely, visual and manual straight-ahead and line bisection. This study gave us the opportunity to explore previously unreported parameters in CRPS. Line bisection is one of the most classical tests used to diagnose spatial neglect [40, 41], which has the advantage of providing continuous measures, unlike cancellation tests, which provide discrete measures. Therefore, it has been shown to be sensitive enough to detect discrete modulations produced in healthy subjects (e.g., [42, 43]). Straight-ahead demonstrations fall in the same category. In neglect patients, the most reliable parameter is the manual straight-ahead [39], found to be reliably deviated toward the healthy side (namely, the ipsilesional side), and this parameter has not yet been reported for CRPS patients, for whom it can be measured for each hand. Interestingly, in case of neglect, visual straight-ahead is usually measured one meter away from the target, whereas it has been measured at two meters in CRPS [11, 12, 29, 44]. Here, we investigated visual straight-ahead both at one and at two meters. In spite of this detailed testing, our data did not reveal a clear pattern of spatial bias resulting from CRPS.

Surprisingly, the visual straight-ahead data at 2 meters, which is the only parameter described to date, did not confirm previous studies. Indeed, our left CRPS patients did not show a significant left deviation, nor did right CRPS showed right deviation, as described in Sumitani et al. [11]. Our data do not confirm Reinersmann et al.'s [12] systematic deviation to the left irrespective of the patients' affected side. In addition, visual straight-ahead at one meter was not significantly deviated by CRPS.

For the manual straight-ahead, right and left CRPS tended to show a deviation of their healthy hand toward the left side, although this trend was not significant. If this trend was to be confirmed in a larger group, it would further confirm the implication of central mechanisms in CRPS.

Line bisections produced by the two hands did not exhibit a significant deviation. This result confirms the lack of bias found for the painful hand by Förderreuther et al. [13]. However, in their study, the right CRPS showed a significant deviation toward the right side when performing bisection with their healthy hand. Taken together, the heterogeneity of the results available for visual straight-ahead and for line bisection shows that CRPS patients may exhibit a great deal of interindividual variability. Although this is also the case in spatial neglect, the magnitude of the expected alterations in CRPS is such that no congruence between studies or a general explicative scheme can be proposed at this stage.

Motor extinction and motor neglect were explored with detailed kinematic recordings.

Motor extinction was explored with a quantitative finger tapping task, designed to check whether patients perform poorer with their affected hand in bimanual condition (motor extinction) and unimanual condition (motor neglect). In addition, a bimanual circle drawing task was used [45] in order to assess whether patients would draw smaller circles with the painful hand than with their healthy one. None of these tasks had previously been reported in CRPS patients. The key prediction resulting from the neglect hypothesis was poorer performance on the affected side, especially in bimanual condition. The finger tapping task in the reference condition (closed eyes) showed no difference between patients and controls (no group effect) or group*∗*condition interaction.

The circle drawing task further explored several aspects of motor extinction and motor neglect. Indexes of ellipse size (perimeter, surface, and horizontal extent) investigated motor extinction while horizontal drift explored motor neglect. Congruently with the previous task analysis, no arguments for motor extinction or motor neglect were found and so patients did not differ from controls in terms of hand asymmetry. In Punt et al.'s bimanual condition [45], a neurological patient with motor neglect drew 65% smaller circles with his affected hand as compared to his healthy hand. Moreover, here, patients draw larger circles than controls and both patients and controls tended to draw smaller circle in full-vision condition.

Altogether, these kinematic results clearly invalidate the motor neglect hypothesis heralded by several authors [14–16, 25] since we demonstrated that no performance difference is observed between healthy hand and painful hand in bimanual condition for these two different tasks.

This study design also offered the opportunity to test how parameters of the circle drawing task and finger tapping task that were not initially affected by CRPS evolved under prism adaptation. The absence of adaptation*∗*hand significant interaction crucially implies that the hand difference observed during the pretest in both patients and controls for the circle task was not affected by the prism adaptation therapy.

In addition, a counterintuitive observation made in both patients and controls in the circle task was that although no hand asymmetry was obtained in the no-vision condition, the left hand tended to draw larger ellipses when more visual feedback was provided. This reliable trend suggests that it is the visual control of the hand movement (and path) that paradoxically contributes to this hand asymmetry. This important result will deserve further investigations in order to disentangle attentional and motor control sources of explanation.

To summarize the spatial cognition examination before intervention, this CRPS patients group did not demonstrate any argument for significant* perceptual* neglect or* motor extinction* or* motor neglect*. Contrary to the classic neglect-like hypothesis, we did not find perceptual neglect-like behavior or antineglect as well, as suggested by Sumitani et al. [11]. Our result does not confirm Reid et al.'s data which showed a tactile bias away from the affected side and a midline bisection bias toward the affected side. The very widespread hypothesis of motor neglect or motor extinction behavior was also refuted by this data. However, some new arguments were raised to corroborate the central participation theory to this syndrome.

### 5.2. Exploring the Functional Outcome of Prism Adaptation Therapy

Our second goal was to explore the concrete effectiveness of prism adaptation which was suggested at several levels. We observed a gradual reduction of initial pain initiated during the adaptation treatment week. This amelioration provided a significantly positive outcome after adaptation that was fully maintained at the follow-up consultation 2 weeks later. This statistical reduction of pain was also clinically relevant because the initial pain (VSA: 58.8 ± 4.8 mm) appeared to stabilize at the follow-up consultation (VSA: 38 ± 8.3 mm), producing an average reduction by about 36% of pain.

To our current knowledge, only two studies have been published: the first by Sumitani et al. [29] with 5 patients and the second by Bultitude and Rafal [35] with only one patient. The pain deficit described in these two studies was about 50% in Sumitani et al.'s study [29] for the five-patient group and about 80% for the single patient described by Bultitude and Rafal [35].

Our group is thus far the largest series (*n* = 7) of CRPS patients undergoing prism adaptation as rehabilitation and the first sample followed up after the end of the rehabilitation period. We managed to follow up all patients between 15 and 20 days after the last adaptation day whereas the longitudinal single case was followed up for 8 weeks without treatment in Sumitani et al.'s study [29] and the patient in Bultitude and Rafal's study [35] followed a two-week-long wash-out period.

One interesting point is that, for both previous patients [29, 35], rapid increasing of pain was described after prism therapy was stopped. Our group study enabled us to suggest that sustainable effects can be produced by a week of intense prism adaptation therapy. Sumitani et al. used one daily session over 14 days, and Bultitude and Rafal extended this period to 3 weeks. Thus, our results indicate a possible dose-effect relation linked to a posology of 2 sessions a day. It seems rather unlikely that the longer treatment period would be responsible for poorer results.

Taking a closer look at the individual data, we noticed that there were good and bad responders: the percentage of individual benefit ranged from 0% to 90% considering the mean pain before and after prism adaptation. Only two patients presented less than 20% of amelioration, one patient presented about 30% of benefit, and a majority of 4 patients presented 40% or more benefits. This outcome is to be confronted with clinical characteristics of our patients sample. Our patients had been showing CRPS for up to 36 months (more than 13 months on average) and most of them had been submitted to at least 3 types of therapeutic interventions. The patients who showed the best benefit were not the least chronic ones: the patient (number 4) with 90% improvement had CRPS for 17 months. Interestingly, the clinical outcome for patients did not seem to be related to the type of CRPS: the two patients with CRPS type 2 had pain score improved by 48% and 10%. This is interesting because so far only type 1 CRPS had been reported to benefit from prism adaptation [29, 35].

Qualitatively, this pain reduction was described by one of the patients producing a drawing of her own hand. This representation showed an impressive global reduction of pain, which decreased from moderate pain concerning nearly the whole hand to no pain at all except for a very tiny weak pain area at follow-up. This reduction occurred with respect to the initial mosaic pattern pain distribution, which did not correspond to any nervous territory/area. Strikingly, improvement even further went on after stopping the prism adaptation therapy. This last point is congruent with the group data analysis showing the persistence of benefits along the last follow-up period and also with the knowledge about prism effect on neglect symptoms [33, 34, 38, 46].

Major information provided by our study deals with the concrete effects of prism adaptation on CRPS patients' quality of life. In this group, the Sickness Impact Profile showed a statistically significant improvement of 26%. Emotional behavior, social interactions, body care and movement, and alertness behavior were particularly concerned. At the individual level, the benefits ranged from 0% to 54%, with 3 patients presenting improvement inferior to 20% and 4 patients between 24% and 54%. Despite being subjective, this evaluation describes objective activities of everyday life and it indicates substantial enhancement of everyday living for this initially disabled group.

Altogether, the very positive outcome of this interventional study on pain and ADL calls for designing larger controlled multicentric studies to assess the clinical stake and the individual responsiveness of chronic and acute CRPS patients. Evidence collected by two previous studies [29, 35] and the present one is sufficient to suggest that repetitive prism adaptation intervention deserves investments to explore its clinical impact on this invalidating disorder. According to our results, intensive adaptation (twice a day) intervention should be preferred to mild (once a day) intervention.

### 5.3. Exploring Frames of Reference in CRPS: Pathophysiology and Physiotherapeutics

Our third goal was to monitor straight-ahead demonstrations throughout prism adaptation therapy, so as to explore their potential causal links with clinical and neuropsychological parameters.

The main result is the confirmation that CRPS patients adapted to prism, as shown by significant sensorimotor posteffects represented by open loop pointing for the adapted hand (OLPAH). Their mean adaptation during the first exposure was 3.6°, that is, smaller than normal subjects undergoing the same amount of deviation (about 7° in [47]). Contrary to our prediction, we did not observe significant evolution of OLPAH between pre- and postintervention, but preadaptation values were significantly modified along the prism adaptation period, suggesting that cumulative effects of this intensive intervention lead to longer-lasting effects than daily sessions of adaptation [29, 35]. Whether this proprioceptive effect altered body representations in such a way as to subsequently modify visual and attentional parameters as it has been shown for spatial neglect [39] remains to be specifically investigated. One hypothesis related to Reid et al.'s discussion [24] is that prism adaptation could play a role in correcting the spatially defined bias in tactile processing away from the affected side and the visuospatial processing bias during midline judgement toward the affected side. Indeed, if Reid et al.'s findings were to be confirmed, they would be coherent with the use of prism adaptation for CRPS. As a matter of fact, the direction of prism adaptation proposed by Sumitani et al. [29] produces manual aftereffects toward the clinically affected side while visual aftereffects lie toward the healthy side; that is, prism adaptation would normalize the two initial biases put forward by Reid et al.

Additional analyses were aimed at exploring the causal link between sensorimotor and clinical measures. No significant correlation was obtained between the sensorimotor aftereffect of the first prism adaptation session and the final gain on pain and quality of life. This is consistent with knowledge about prism adaptation clinic efficiency on neglect after stroke, which has never proved to be correlated with the magnitude of sensorimotor aftereffects [38, 48].

## 6. Conclusion

In brief, this study provided detailed analysis of the largest CRPS group before, during, and following an intense prism adaptation intervention. At the pathophysiological level, patients' spatial cognition was explored using various sensitive and quantitative tools and providing no systematic left-right asymmetry pattern, in sharp contrast to the clear expectations expressed in the literature. At the therapeutic level, prism adaptation is confirmed to be a very promising method to alleviate CRPS pain. Our study reveals that intense prism adaptation intervention produces sustainable therapeutic effects on pain and subsequent benefits on quality of life.

It has now become timely to design and realize controlled trials to test the effectiveness and durability of this promising, intense prism adaptation intervention, to explore predictive parameters of patients' responsiveness and neurophysiological mechanisms of this therapeutic effect. Ultimately tailoring interventions on the basis of CRPS subgroups [7] is a key to future routine care of this intriguing chronic pain condition.

## Figures and Tables

**Table 1:** Patients' description.

Patient	Gender	Age	Dominant hand	Type	Affected side	Disease duration (months)	Mean pain intensity (VAS 0–100) over the last week	Treatment	Previous care
Ms. H.	F	50	R	1	L	36 M	50	(i) Paracetamol (ii) Amitriptyline (iii) Paroxetine (iv) Alprazolam	(i) Physiotherapy + occupational therapy (ii) Analgesics, levels 1, 2, and 3 (iii) TENS

Ms. F.	F	51	R	1	L	4 M	75	(i) Lidocaine patch (ii) Flavonoid fraction (iii) Amitriptyline	Analgesic only

Mr. D.	M	30	R	2	R	6 M	70	(i) Morphine sulfate (ii) Nefopam (iii) Paracetamol (iv) Clobazam (v) Gabapentin	(i) Mirror therapy (ii) Physiotherapy + occupational therapy

Ms. A.	F	55	R	1	R	17 M	38.5	(i) Duloxetine (ii) Paracetamol (iii) Zopiclone	(i) Physiotherapy + occupational therapy (ii) Mirror therapy (iii) Sympathetic blockades × 6

Ms. dCS.	F	24	R	1	R	4 M	55	(i) Amitriptyline (ii) Paracetamol (iii) Lidocaine patch (iv) Phytotherapy	(i) Physiotherapy (ii) TENS

Ms. O.	F	43	R	1	R	20 M	65	(i) Paracetamol (ii) Codeine	(i) Physiotherapy (ii) TENS (iii) Specialized follow-up

Ms. C.	F	58	R	1	R	6 M	55	(i) Paracetamol (ii) Tramadol (iii) Amitriptyline	(i) Physiotherapy

There were 6 women and one man, aged from 24 to 58. Five patients presented CRPS type 1 and two patients presented CRPS type 2. They all underwent various care before inclusion.

**Table 2:** Spatial reference frame deviation for patients at inclusion and before the first adaptation (mean and standard error of the mean).

	Right CRPS (*n* = 5)				Left CRPS (*n* = 20)			
	Inclusion		Pre-ADA1		Inclusion		Pre-ADA1	
	Mean	SEM	Mean	SEM	Mean	SEM	Mean	SEM
VSA1m	0.1	2.3	−2.7	2	1.5	0	−1.4	5.6
VSA2m	−2.1	1	0.6	2	0.6	0.5	−0.2	0.1
RMSA	0.6	1.8	−0.5	2.2	−5.9	0.4	−1.5	3.2
LMSA	4.1	2	−4.1	2.1	−0.3	2.6	−2.2	8.3
RLB (cm)	0.5	0.8	1.6	1.7	−2.35	5.9	−2.35	5.9
LLB (cm)	0.7	0.7	−1	0.6	−4.5	8.8	−6.6	10.5

Pre-ADA1: pretest performed prior to the first prism adaptation session; VSA1m: visual straight-ahead at one meter; VSA2m: visual straight-ahead at two meters; RMSA: right manual straight-ahead; LMSA: left manual straight-ahead.

**Table 3:** Data description for finger tapping task for controls and patients, before and after adaptation.

	Controls				Preadaptation patients				Postadaptation patients			
	Left hand		Right hand		Affected hand		Healthy hand		Affected hand		Healthy hand	
	Means	SEM	Means	SEM	Means	SEM	Means	SEM	Means	SEM	Means	SEM
No vision												
Period (ms)												
Uni	**447.5**	*22.1*	**441.2**	*22.4*	**482.6**	*32.9*	**464.9**	*20.2*	**477.6**	*11.4*	**471.7**	*12.2*
Bi	**437.4**	*24.4*	**437.2**	*24.4*	**474.9**	*26.9*	**472.1**	*24.8*	**471.8**	*10.4*	**471.7**	*10.5*
Amplitude (mm)												
Uni	**42.7**	*4.7*	**37.5**	*3.4*	**29.6**	*5.9*	**35.9**	*5.6*	**28.2**	*5.3*	**36.4**	*3.8*
Bi	**37.9**	*3.4*	**31.2**	*1.3*	**29.4**	*4.7*	**34.5**	*4.7*	**27.2**	*4.5*	**32.6**	*3.5*

Crossed hands												
Period (ms)												
Uni	**439.8**	*26.5*	**440.5**	*25.2*	**472.6**	*25.8*	**467.3**	*19.7*	**470.9**	*15.6*	**463.2**	*16.6*
Bi	**438.2**	*27.1*	**438.1**	*27.3*	**481.6**	*30.2*	**471.8**	*22.0*	**465.6**	*13.0*	**465.4**	*12.9*
Amplitude (mm)												
Uni	**39.7**	*2.1*	**34.2**	*2.2*	**27.8**	*4.3*	**33.0**	*4.2*	**26.2**	*5.0*	**36.1**	*3.3*
Bi	**39.1**	*2.2*	**31.2**	*0.8*	**26.8**	*4.4*	**32.1**	*4.4*	**26.1**	*3.2*	**32.9**	*2.3*

Full vision												
Period (ms)												
Uni	**449.1**	*12.4*	**450.4**	*12.7*	**474.4**	*26.0*	**452.4**	*23.1*	**465.4**	*11.9*	**466.5**	*11.0*
Bi	**446.0**	*15.4*	**445.4**	*15.3*	**455.4**	*26.1*	**454.7**	*24.9*	**464.0**	*9.4*	**464.1**	*9.5*
Amplitude (mm)												
Uni	**43.7**	*3.2*	**38.5**	*2.4*	**35.6**	*5.9*	**37.2**	*4.7*	**30.2**	*6.0*	**37.3**	*3.6*
Bi	**43.7**	*3.6*	**37.9**	*2.1*	**32.8**	*5.5*	**36.8**	*4.8*	**29.8**	*4.7*	**37.8**	*2.4*

Mean and standard error of the mean for the three conditions (no vision, crossed hands, and full vision) are represented.

**Table 4:** Comparison between patients before adaptation and controls for the three conditions used in the tapping task.

	Effect	Group		Condition (uni/bi)		Hand		Group × condition		Group × hand		Condition × hand		Group × condition × hand	
ANOVA	Statistics	*F*(1,9)	*p*	*F*(1,9)	*p*	*F*(1,9)	*p*	*F*(1,9)	*p*	*F*(1,9)	*p*	*F*(1,9)	*p*	*F*(1,9)	*p*
No vision	Period	1.16	0.310	4.69	0.059	0.20	0.666	1.66	0.230	2.46	0.152	0.13	0.731	2.88	0.124
	Amplitude	1.18	0.306	7.92	**0.020**	4.78	0.057	2.15	0.176	0.35	0.569	0.36	0.563	0.55	0.477

Crossed hands	Period	0.83	0.386	0.00	0.956	0.71	0.423	2.09	0.182	0.70	0.425	0.09	0.765	0.00	0.968
	Amplitude	2.88	0.124	5.60	**0.042**	8.50	**0.017**	0.11	0.742	0.26	0.624	1.53	0.247	0.40	0.541

Full vision	Period	0.45	0.520	3.94	0.078	2.42	0.154	0.05	0.822	2.24	0.169	2.82	0.128	1.68	0.227
	Amplitude	1.73	0.221	0.28	0.612	5.75	**0.040**	0.05	0.825	0.31	0.590	0.15	0.705	0.02	0.883

Crucially, no main effect of group or group *∗* hand interaction was found.

**Table 5:** Statistical analysis of the prism adaptation effects found in CRPS patients for the finger tapping task.

	Effect	Adaptation (pre/post)		Condition (uni/bi)		Hand (affected/healthy)		Adaptation × condition		Adaptation × hand		Adaptation × hand		Adaptation × condition × hand	
ANOVA	Statistics	*F*(1,4)	*p*	*F*(1,4)	*p*	*F*(1,4)	*p*	*F*(1,4)	*p*	*F*(1,4)	*p*	*F*(1,4)	*p*	*F*(1,4)	*p*
No vision	Period	0.00	0.981	0.70	0.449	0.90	0.396	0.37	0.578	0.50	0.518	0.93	0.389	0.43	0.546
	Amplitude	0.48	0.527	2.73	0.174	5.18	0.085	5.01	0.089	0.17	0.701	3.65	0.129	2.11	0.220

Crossed hands	Period	0.25	0.646	1.18	0.338	0.57	0.492	1.26	0.325	0.22	0.662	0.12	0.746	1.46	0.294
	Amplitude	0.03	0.865	2.57	0.184	4.80	0.094	0.10	0.773	2.24	0.209	1.06	0.361	4.13	0.112

Full vision	Period	0.12	0.746	5.52	0.079	1.77	0.255	1.41	0.301	1.84	0.247	1.82	0.249	1.73	0.259
	Amplitude	0.36	0.583	0.71	0.448	3.99	0.116	1.44	0.296	3.87	0.121	2.05	0.226	0.58	0.489

No significant effect was found for adaption, condition, and hand or crucially adaptation *∗* hand interaction.

**Table 6:** Results of the circle drawing task obtained in the patients group before and after adaptation and in the control group.

	Controls				Preadaptation patients				Postadaptation patients			
	Left hand		Right hand		Healthy hand		Affected hand		Healthy hand		Affected hand	
	Mean	SEM	Mean	SEM	Mean	SEM	Mean	SEM	Mean	SEM	Mean	SEM
No vision												
Perimeter	**462.6**	*15.0*	**451.4**	*12.2*	**490**	*43.7*	**489.6**	*37.5*	**543.9**	*19.4*	**545.4**	*10.9*
Angle	**11.2**	*4.2*	−4.3	*4.0*	**1.0**	*3.2*	**3.9**	*4.6*	**7.5**	*5.7*	**1.1**	*5.4*
H. drift	−6.8	*4.1*	**9.6**	*2.4*	−7.0	*3.8*	**16.3**	*4.9*	−12.3	*4.4*	**18.0**	*3.4*
H. extent	**131.7**	*5.1*	**127.4**	*4.4*	**141.8**	*11.2*	**136.5**	*7.5*	**152.7**	*4.9*	**149.4**	*5.9*
Surface	**15978**	*906*	**15582**	*747*	**19271**	*3516*	**18785**	*2896*	**22676**	*1624*	**22591**	*905*

Hand vision												
Perimeter	**454.4**	*12.7*	**453.6**	*9.1*	**489**	*46.8*	**474.5**	*40.7*	**538.6**	*24.8*	**524.3**	*18.8*
Angle	−4.6	*3.2*	**6.2**	*4.0*	−2.9	*5.6*	**6.6**	*1.5*	−5.2	*5.3*	**5.3**	*2.4*
H. drift	**1.7**	*4.6*	**11.3**	*3.1*	**1.5**	*4.1*	**9.3**	*2.4*	**2.3**	*3.4*	**11.6**	*4.4*
H. extent	**125.5**	*4.0*	**125.6**	*5.0*	**136.5**	*10.2*	**132.2**	*8.7*	**151.3**	*6.6*	**147.6**	*4.6*
Surface	**15603**	*764*	**15621**	*562*	**18748**	*3480*	**17736**	*2958*	**22145**	*1943*	**21234**	*1438*

Full vision												
Perimeter	**398.2**	*19.2*	**376.2**	*20.7*	**471.6**	*25.0*	**434.6**	*22.5*	**516.8**	*23.8*	**485.4**	*26.9*
Angle	**6.4**	*1.8*	−5.4	*3.1*	**3.9**	*3.0*	−4.8	*3.9*	−0.3	*3.3*	−1.0	*4.8*
H. drift	**2.7**	*3.2*	−3.6	*2.3*	−4.3	*3.5*	**1.3**	*4.3*	−3.9	*3.3*	**2.5**	*3.0*
H. extent	**119.1**	*6.0*	**108.1**	*6.2*	**138.2**	*6.8*	**128.8**	*6.2*	**145.9**	*4.7*	**132.4**	*7.4*
Surface	**12100**	*1058*	**11002**	*1036*	**17019**	*1729*	**14613**	*1423*	**20229**	*1841*	**17909**	*2032*

The main pre-post effect did not lead to significant results in any of the three conditions. Additionally, no pre-post contribution to interaction effects was obtained. Several hand and direction main effects were observed as in the previous analysis comparing patients to controls.

**Table 7:** Comparison of the circle drawing task results obtained in patients and controls before adaptation.

	Effects	Group		Hand		Direction		Group × hand		Hand × direction		Group × direction		Group × hand × direction	
ANOVA	Statistics	*F*(1,10)	*p*	*F*(1,10)	*p*	*F*(1,10)	*p*	*F*(1,10)	*p*	*F*(1,10)	*p*	*F*(1,10)	*p*	*F*(1,10)	*p*
No vision	Perimeter	0.50	0.493	0.13	0.721	11.70	**0.006**	0.34	0.570	5.87	**0.036**	0.09	0.763	0.03	0.860
	Angle	0.04	0.84	6.61	**0.03**	0.07	0.790	0.87	0.373	12.30	**0.005**	0.32	0.584	2.16	0.172
	Horiz. drift	0.69	0.425	19.00	**0.001**	2.90	0.119	0.57	0.467	1.45	0.256	0.28	0.61	1.07	0.324
	Horiz. extent	1.09	0.32	1.53	0.244	15.30	**0.002**	0.13	0.720	0.03	0.857	0.87	0.372	0.49	0.499
	Surface	0.82	0.386	0.28	0.610	11.27	0.007	0.00	0.986	1.53	0.244	0.01	0.962	0.34	0.571

Hand vision	Perimeter	0.32	0.583	0.31	0.588	10.77	**0.008**	0.25	0.627	0.89	0.361	0.01	0.941	1.61	0.233
	Angle	0.10	0.755	3.21	0.103	2.50	0.144	0.06	0.811	0.01	0.902	0.43	0.526	0.61	0.452
	Horiz. drift	0.09	0.763	7.10	**0.023**	9.24	**0.012**	0.03	0.850	1.28	0.284	0.03	0.87	0.56	0.471
	Horiz. extent	0.95	0.350	0.39	0.545	49.26	**0.001**	0.16	0.695	0.01	0.921	0.01	0.914	0.00	0.970
	Surface	0.55	0.476	0.40	0.539	14.44	**0.003**	0.48	0.503	0.34	0.569	0.01	0.991	0.71	0.420

Full vision	Perimeter	3.85	0.078	23.47	**0.001**	0.09	0.761	1.97	0.190	0.88	0.370	0.66	0.435	0.32	0.584
	Angle	0.07	0.793	10.26	**0.009**	0.13	0.726	0.02	0.883	7.28	**0.022**	0.21	0.653	0.61	0.454
	Horiz. drift	0.22	0.649	0.02	0.887	0.67	0.431	2.37	0.155	2.00	0.187	0.82	0.387	0.195	0.668
	Horiz. extent	5.30	**0.044**	18.68	**0.001**	0.84	0.381	0.18	0.681	1.03	0.333	0.03	0.859	0.65	0.439
	Surface	4.35	0.063	19.59	**0.001**	0.67	0.431	3.30	0.099	1.16	0.307	1.12	0.315	0.32	0.583

In the reference no-vision condition, no group effect was observed. A significant hand effect logically affected the horizontal drift (the two hands symmetrically drifting apart in order not to bump each other). The ellipse main axis angle was also logically affected by a hand *∗* direction interaction as a result of biomechanical constraints. More interestingly, no group effect was observed, neither as a main effect nor as interaction effects.

**Table 8:** Statistical analysis of patients performance before and after prism adaptation in the circle drawing task.

	Effects	Pre/post		Hand		Direction		Pre/post × hand		Hand × direction		Pre/post × direction		Pre/post × hand × direction	
ANOVA	Statistics	*F*(1,5)	*p*	*F*(1,5)	*p*	*F*(1,5)	*p*	*F*(1,5)	*p*	*F*(1,5)	*p*	*F*(1,5)	*p*	*F*(1,5)	*p*
No vision	Perimeter	2.84	*0.152*	0.00	*0.978*	39.54	***0.001***	1.26	*0.311*	1.42	*0.286*	15.48	***0.011***	0.51	*0.508*
	Angle	0.38	*0.564*	4.50	*0.087*	210.9	***0.000***	4.18	*0.096*	0.00	*0.971*	2.23	*0.195*	1.65	*0.255*
	Horiz. drift	0.69	*0.443*	6.66	***0.049***	2.28	*0.191*	0.02	*0.896*	2.23	*0.195*	1.43	*0.285*	0.01	*0.913*
	Horiz. extent	1.33	*0.300*	0.94	*0.377*	16.04	***0.010***	0.01	*0.935*	0.134	*0.729*	3.53	*0.119*	0.01	*0.935*
	Surface	2.05	*0.211*	0.24	*0.646*	31.21	***0.002***	0.29	*0.612*	0.01	*0.924*	3.16	*0.136*	0.47	*0.525*

Hand vision	Perimeter	2.78	*0.156*	0.71	*0.438*	16.86	***0.003***	0.00	*0.960*	0.17	*0.698*	0.41	*0.551*	5.15	*0.072*
	Angle	1.62	*0.259*	1.31	*0.304*	1.33	*0.301*	0.28	*0.620*	2.36	*0.185*	0.74	*0.43*	0.00	*0.960*
	Horiz. drift	0.66	*0.453*	3.20	*0.133*	3.81	*0.108*	0.89	*0.388*	2.93	*0.147*	0.95	*0.375*	0.55	*0.489*
	Horiz. extent	3.07	*0.140*	0.71	*0.438*	66.62	***0.001***	0.00	*1.000*	0.04	*0.843*	0.299	*0.608*	0.06	*0.805*
	Surface	2.43	*0.18*	0.80	*0.412*	33.11	***0.002***	0.15	*0.716*	0.01	*0.921*	0.94	*0.376*	1.53	*0.270*

Full vision	Perimeter	5.05	*0.075*	12.65	***0.016***	0.09	*0.770*	0.20	*0.674*	0.63	*0.463*	0.95	*0.375*	0.44	*0.534*
	Angle	0.14	*0.725*	1.67	*0.255*	0.58	*0.479*	0.80	*0.411*	3.74	*0.11*	0.130	*0.733*	1.30	*0.305*
	Horiz. drift	0.73	*0.430*	1.03	*0.357*	1.11	*0.340*	0.14	*0.720*	2.08	*0.209*	0.01	*0.916*	0.89	*0.389*
	Horiz. extent	0.54	*0.495*	7.89	***0.037***	1.15	*0.333*	4.50	*0.087*	0.33	*0.591*	0.17	*0.693*	2.03	*0.214*
	Surface	4.62	*0.084*	12.00	***0.018***	5.85	*0.060*	0.06	*0.809*	0.00	*0.933*	0.04	*0.841*	0.13	*0.728*

Crucially no significant pre/post *∗* hand interaction was obtained, which means that the hand difference observed during the pretest in patients and controls was not affected by the prism adaptation therapy. Only hand and direction effects reached significance, confirming the main effects found during the pretest in both subjects groups. The hand effects tended to be prominent in the full-vision condition whereas the direction effects tended to be prominent in the no-vision condition.
